# Ruptured Granulosa Cell Tumor of the Ovary as a Cause of Acute Abdomen in Postmenopausal Woman

**DOI:** 10.1155/2012/451631

**Published:** 2012-09-03

**Authors:** Tufan Oge, S. Sinan Ozalp, Omer T. Yalcin, Sare Kabukcuoglu, Emine Arslan

**Affiliations:** ^1^Department of Obstetrics and Gynecology, Eskisehir Osmangazi University School of Medicine, 26100 Eskisehir, Turkey; ^2^Department of Pathology, Eskisehir Osmangazi University School of Medicine, Eskisehir, Turkey

## Abstract

Acute abdomen with hemoperitoneum is a very rare entity in postmenopausal women due to gynecologic conditions. A 54-year-old, postmenopausal woman was brought to emergency department with severe abdominal pain. Physical examination revealed acute abdomen findings with 15 cm pelvic mass on the right adnexal region. Immediate exploratory laparotomy was performed. During laparotomy 1000 cc of bloodstained fluid, ruptured and actively bleeding large mass arising from right ovary was observed. Right salpingo-oopherectomy was performed in emergency conditions, and pathology report revealed an adult type of granulosa cell tumor. After this result, staging surgery was performed and patient was diagnosed as granulosa cell tumor stage 1 c. Cisplatin, etoposide, and bleomycin chemotherapy was given. Clinicians should be aware of granulosa cell tumors which may occur at any age and prone to rupture. Frozen section will be helpful in order to avoid incomplete surgeries especially in postmenopausal women presented with intra-abdominal bleeding.

## 1. Introduction

Granulosa cell tumors of the ovary are rare neoplasms that originate from sex-cord stromal cells and they comprise 2–5% of all ovarian cancers [1]. Women may present with an asymptomatic mass or symptoms related to hyperestrogenism like abnormal uterine bleeding, breast tenderness, and postmenopausal bleeding in adult form of the disease. Although acute abdomen with hemoperitoneum is a common gynecologic emergency, it is a very rare entity in postmenopausal women due to gynecologic conditions. Lee et al. reported that tumor rupture occurred in 17.6% of cases before surgery diagnosed as adult granulosa cell tumor but the pain did not present as a cause of acute abdomen [1]. To the best of our knowledge, there are four cases of hemoperitoneum because of ruptured granulosa cell tumor [2–5]. We report a 54-year-old postmenopausal woman who presented with acute abdomen and hemoperitoneum because of ruptured granulosa cell tumor.

## 2. Case Report

A 54-year-old, gravida 3, para 2 woman was brought to emergency department with severe right lower abdominal pain. The patient who was at menopause for 2 years had a postmenopausal bleeding history for two months, and she had abdominal distension for three months. Physical examination revealed abdominal tenderness and acute abdomen findings with stable vital signs, and pelvic examination revealed nearly 15 cm pelvic mass on the right adnexal region. Her hemoglobin count was 11.7 gr/dl. Abdominal ultrasound and pelviabdominal tomography examination confirmed the mass in the right adnexa measuring 13 × 12 × 8 cm with multiple septations and also revealed free fluid at Morisson's space and the cul-de-sac. Because of the clinical findings and 1 gr/dl hemoglobin fall in three hours, an immediate exploratory laparotomy was performed. During laparotomy 1000 cc of bloodstained fluid, ruptured and actively bleeding large mass arising from right ovary was observed. Omentum, the left ovary, and the uterus were normal, and right salpingo-oopherectomy was performed in emergency conditions. Postoperative recovery was uneventful, and the patient was discharged from hospital after endometrial sampling was performed because of patient's history of postmenopausal bleeding. Pathology report revealed an adult type of granulosa cell tumor, the mitotic rate was 9 mitoses per 10 HPFs (high power fields), and endometrial biopsy result was complex hyperplasia. According to this result, the patient was evaluated as granulosa cell tumor at least stage 1 c, and staging surgery with type II hysterectomy + left unilateral salpingo-oopherectomy + infracolic omentectomy + bilateral pelvic-para-aortic lymphadenectomy + fluid sampling for cytologic examination was performed. Patient was finally diagnosed as granulosa cell tumor stage 1 c according to the pathology result of staging surgery and 6 cycles of cisplating + etoposid + bleomycin chemotherapy was given.

## 3. Discussion

Abdominal pain is the most common surgical emergency, the most common cause for a surgical consultation in the emergency department, and the most common cause for nontrauma-related hospital admissions [6]. The differential diagnosis of acute abdomen is wide, ranging from benign to life-threatening conditions. Elderly patients often have vague, nonspecific complaints and atypical presentations of potentially life-threatening conditions leading to time-consuming workups. Older patients with abdominal pain have a six-to-eightfold increase in mortality compared to younger patients. The elderly account for 20 percent of emergency department visits, of which three to four percent are for abdominal pain. About one-half to two-thirds of these patients require hospitalization, while one-third require surgical intervention [7].

Gynecologic conditions still cause acute abdomen in postmenopausal women. As the number of postmenopausal women increases, physicians will have more opportunities to treat elderly women with gynecological complications. The gynecologic causes of acute abdomen in postmenopausal women can be adnexal torsion, pelvic abscess, pyosalpinx, and hemoperitoneum [8–10]. Ovarian torsion and rupture causes severe abdominal pain. In postmenopausal women, ovarian masses large enough to suffer torsion must be considered malignant until proven benign. In addition causes of hemoperitoneum must be taken into consideration including ovarian bleeding due to anticoagulant therapy or retrograde bleeding due to a stenotic cervix in women receiving hormonal therapy. In many cases laparotomy is needed for differential diagnose, and in the present case during exploration hemoperitoneum and active bleeding ovarian mass is noticed.

Clinicians should be aware of granulosa cell tumors which may occur at any age and prone to rupture. Frozen section will be helpful in order to avoid incomplete surgeries in malign conditions especially in postmenopausal women presented with intra-abdominal bleeding.
